# YTHDF3 Is Involved in the Diapause Process of Bivoltine *Bombyx mori* Strains by Regulating the Expression of *Cyp307a1* and *Cyp18a1* Genes in the Ecdysone Synthesis Pathway

**DOI:** 10.3390/biom12081127

**Published:** 2022-08-17

**Authors:** Yanhua Chen, Bingyan Fan, Ayinuer Yasen, Juan Zhu, Meixian Wang, Xingjia Shen

**Affiliations:** 1 Jiangsu Key Laboratory of Sericultural Biology and Biotechnology, Jiangsu University of Science and Technology, Zhenjiang 212100, China; 2 Key Laboratory of Silkworm and Mulberry Genetic Improvement, Ministry of Agriculture and Rural Affairs, Sericulture Research Institute, Chinese Academy of Agricultural Sciences, Zhenjiang 212100, China

**Keywords:** *Bombyx mori*, diapause, YTHDF3, RNA N6-methyladenosine, *Cyp307a1*, *Cyp18a1*, mRNA stability, translation

## Abstract

The variable diapause features of bivoltine silkworm (*Bombyx mori*) strains regulated by environmental signals in the embryonic stage are closely related to epigenetics. Previously, we showed that the expression of YTHDF3 is significantly different in the pupae of the bivoltine silkworm Qiufeng developed from eggs incubated at a normal temperature (QFHT, diapause egg producer) compared to those from eggs incubated at a low temperature (QFLT, nondiapause egg producer), indicating that the expression of diapause-associated genes is regulated by the m^6^A modification level. However, how YTHDF3 regulates the expression of diapause-related genes remains unclear. In this study, we observed that the knockdown of *B. mori* YTHDF3 resulted in delayed embryo development, while the overexpression of YTHDF3 resulted in the transformation of nondiapause-destined eggs into a mixture of diapause and nondiapause eggs. Further studies showed that YTHDF3, as a reading protein, can recognize the m^6^A site of *Cyp307a1* and *Cyp18a1* genes in the ecdysone synthesis pathway (ESP), and the overexpression of YTHDF3 affects the diapause traits of the silkworm by decreasing the stabilities of mRNAs of *Cyp307a1* and *Cyp18a1* and inhibiting their translation. The above results demonstrate that m^6^A modification mediates YTHDF3 to affect the expression levels of its target genes, *Cyp307a1* and *Cyp18a1*, in the ESP to regulate diapause in bivoltine *B. mori*. This is the first report of the m^6^A methylation regulation mechanism in diapause in *B. mori* and provides new experimental data for clarifying the diapause regulation network.

## 1. Introduction

The N6-adenosine methylation (m^6^A) modification is the most common internal modification of eukaryotic mRNAs and is involved in the regulation of various physiological processes, such as growth and development, neural activity and stress response [1,2,3]. It directs the beginning of mRNAs’ different fates by differentially modifying the target mRNAs, while the recognition and initiation of the downstream pathway by readers is the key to the function of m^6^A modification. Different readers have different localizations in cells and have a specific recognition mechanism, which affects the splicing, transportation, stability and translation efficiency of modified RNAs [4,5,6,7]. RNA m^6^A methylation modifications are widespread in organisms, but the number of methylated readers that have been identified thus far is limited. Studies have shown that readers mainly include the HNRNP family [8,9], IGF2BP [10], eIF3 [11] and the YTH family. The YTH family is currently the most well-studied family [12,13,14]. YTHDF1, -2 and -3 are homologues in the YTH family that can directly recognize m^6^A modification sites. Although the three YTHDF proteins share high homology, their respective functions are different. They bind to m^6^A modification sites in a substantially similar manner and mediate the fate of the same mRNA in a redundant manner [15]. However, the mechanism of the functional differences among the three YTHDFs remains unclear.

The formation of dormancy or diapause often requires the induction of environmental signals [16,17,18], during which epigenetics is the proposed response to environmental signals and the regulation of phenotypes. Complex epigenetic modifications coordinately regulate transcription and translation in the body through multiple enhancement signals, including histone modifications, DNA modifications, RNA modifications and transcription factors. It is a heritable variation that does not depend on changes in gene sequence [19]. Studies have shown that epigenetics is involved in some important links in the regulation of insect diapause, such as DNA methylation that can respond to photoperiod signals [20,21], histone acetylation that affects insect metamorphosis and development [22] and histone acetylation and methylation modifications that are also involved in the regulation of diapause-related genes in insects [23,24].

The diapause traits of the bivoltine silkworm (*Bombyx mori*) strain are regulated by environmental factors, including temperature and the rhythm of light, especially in the early embryonic stage of the parents. This is a typical epigenetic modification. Studies have shown that the expression level of DNA methyltransferase in silkworm diapause eggs is higher, which is speculated to be involved in the maintenance of silkworm egg diapause [25]. The expression of the DNA methyltransferase genes *BmDnmt*1 and *BmDnmt*2 can be induced by hydrochloric acid treatment of bivoltine silkworm strains, and hydrochloric acid treatment can terminate the diapause of silkworm eggs in the early embryonic stage [26].

Studies on epigenetic modification in insect diapause regulation at the RNA level are very limited, and only a few studies have been reported of *B. mori*. In different silkworm strains, methylation-related enzyme genes showed different expression patterns in different tissues, and there were significant differences in the m^6^A modification of total RNA in the ovarian tissues of bivoltine silkworm pupae from eggs incubated at a normal temperature (QFHT, diapause egg producer) compared to those from eggs incubated at a low temperature (QFLT, nondiapause egg producer). The difference in the expression level of YTHDF3 among m^6^A modification-related enzymes is the highest, consistent with the difference in m^6^A modification, indicating that m^6^A modification may regulate the expression of diapause-related genes in the bivoltine silkworm [27]. However, the mechanism by which m^6^A regulates bivoltine silkworm diapause is still unclear.

Based on the fact that m^6^A modification in the bivoltine silkworm can regulate the expression of diapause-related genes, we speculate that reader protein can regulate the expression of diapause-related genes in bivoltine silkworm and changes their diapause traits. To validate this speculation, in this experiment, we first knocked down YTHDF3 by RNAi in 2-day-old pupae of QFLT and observed the delayed development of eggs compared with the control. Then, the recombinant baculovirus expression vector with YTHDF3 was constructed and injected into the pupae of 2-day-old QFLT for the overexpression of YTHDF3, and the moths laid diapause and nondiapause mixed eggs after crossing, indicating that YTHDF3 is involved in the regulation of diapause. The function of YTHDF3 as a reader protein is to affect the level of gene expression. Third, based on MeRIP-seq, we further verified that the m^6^A methylation sites of *Cyp307a1* and *Cyp18a1* could be recognized and regulated by YTHDF3. *Cyp307a1* and *Cyp18a1* are two important genes in the ESP pathway that regulate the expression of 20,26-dihydroxyecdysis genes and affect the diapause of *B. mori*. Overexpression of YTHDF3 reduced the mRNA stabilities of *Cyp307a1* and *Cyp18a1* and inhibited their translation to affect the diapause of the silkworm. In conclusion, environmental factors (mainly temperature) during the incubation of bivoltine silkworm eggs affect the expression level of YTHDF3 in a certain way and regulate the formation of diapause traits by degrading or inhibiting the expression of the *Cyp307a1* and *Cyp18a1* genes in the ESP.

## 2. Materials and Methods

### 2.1. Animals

A bivoltine strain of *B.* *mori* Qiufeng was used in the experiment. According to the principle that diapause of the bivoltine strain is regulated by environmental factors, mainly temperature and light, diapause-terminated silkworm egg batches (one batch produced by one female moth) were divided into two semibatch groups, one of which was incubated at 17 °C in darkness (QFLT) to produce nondiapause eggs. To produce diapause eggs, the other sample group was incubated at 25 °C under a natural day/night cycle (QFHT) 15 days later to hatch on the same day. After hatching, the larvae of both groups were raised with fresh mulberry leaves under 25 °C with a relative humidity of 80% ± 5% under natural light. Ovary tissue was taken for the samples from 1 day to 6 days from pupae stored at −80 °C for later use. Five pupae were taken as one sample, and each sample was replicated in three groups.

### 2.2. Reagents

The pFastBac dual vector, pGL-*A3*-*luc*-sv40 vector and BmBacJS13 were provided by the Key Laboratory of Sericultural Research Institute, Chinese Academy of Agricultural Sciences (CAAS). Primer synthesis and sequencing were performed by Sangon Biotech (China). RIPA Lysis Buffer (Strong), SDS–PAGE Gel Kit (CWBIO), One Step Western Kit HRP (Mouse) and TC-100 were purchased from Applichem (Germany), and foetal bovine serum was purchased from Corning (USA). The plasmid extraction kit, RT-PCR kit and SYBR Green PCR kit were obtained from Vazyme Company.

### 2.3. Cell Culture

*B. mori* ovary-delivered BmN cells were cultured in TC-100 insect medium supplemented with 10% FBS and 1% penicillin–streptomycin. Plasmid transfection was achieved using Effectene Transfection Reagent (Qiagen) according to the manufacturer’s protocol. Overall, 6 h after transfection with plasmid or dsRNA, the FBS medium was changed to a medium with 10% FBS and cultured continually for another 48 h to observe the fluorescence or to collect cells.

### 2.4. Construction of the Baculovirus Expression System

Primers for *egfp* and *YTHDF3* were designed as listed in Table 1. The *egfp* was inserted into the sites between *BamH*I and *EcoR*I downstream of the pH promoter, and *YTHDF3* was inserted into the site between *EcoR*I and *Xba*I downstream of the pFastBac Dual-egfp vector. The constructed pFastBac Dual-*egfp* and pFastBac Dual-*egfp*-*YTHDF3* plasmids were transformed into BmBacJS13 competent cells to construct recombinant baculoviruses BmBacJS13-*egfp* and BmBacJS13-*egfp*-*YTHDF3*.

### 2.5. dsRNA Synthesis

The dsRNA primers of *YTHDF3* were designed according to the NCBI database and the online software SnapDragon—dsRNA Design (Table 1). After PCR with the two pairs of primers, the T7 Megascript Kit (NEB) was used to prepare the template for PCR of dsRNA, and the double-linked dsRNA was synthesized to achieve knockdown.

### 2.6. In Vivo Injection

In total, 2 μL of dsRNA and 1 μL of recombinant baculovirus were injected into 2-day-old pupae of QFLT with a microinjector, respectively. The injected pupae were preserved at 25 °C until incubation. Then, the female and male moths were crossed for 4 h to produce eggs for phenotypic observation, including egg colour, size and other diapause-related traits.

### 2.7. RIP: RNA Immunoprecipitation

Cells infected with BmDH10Bac-*egfp* and BmDH10Bac-*egfp*-*YTHDF3* were washed with PBS and then collected and centrifuged. Cells were resuspended in cell lysate supplemented with protease inhibitors and ribonuclease inhibitors and incubated on ice for 30 min. Then, 1.5 µg of EGFP antibody or control IgG was conjugated to Protein A/G Magnetic Beads by incubation for 4 h at 4 °C, followed by 3× washing and incubation with precleared nuclear extraction in RIP buffer (150 mM KCl, 25 mM Tris (pH 7.4), 5 mM EDTA, 0.5 mM DTT, 0.5% NP40, 1× protease inhibitor, 100 U/mL ribonuclease inhibitors) at 4 °C overnight. After washing with RIP buffer three times, the beads were resuspended and digested in 100 μL of PBS with 30 μg of Proteinase K at 37 °C for 15 min. RNA was extracted using TRIzol reagent.

### 2.8. RNA Isolation and Quantitative RT-PCR Analysis

After RNA extraction with TRIzol reagent, reverse transcription was performed using a reverse transcription kit according to the manufacturer’s protocol and quantified by real-time PCR using Power SYBR Green Master Mix for relative quantitative analysis of the expression levels of the relevant genes. The primers are shown in Table 1.

### 2.9. mRNA Stability Assay

Cells were seeded in six-well plates, and YTHDF3 was overexpressed by infection with recombinant baculovirus. Then, the cells were treated with 5 μg/mL of actinomycin D to block transcription and collected at various time points, and total RNA was extracted and subjected to RT-qPCR to analyze the stability of the target RNAs.

Since actinomycin D treatment results in transcription stalling, the change in mRNA concentration at a given time (dC/dt) is proportional to the constant of mRNA decay (K) and mRNA concentration (C), leading to the following equation: dC/dt = −KC, thus the mRNA degradation rate K was estimated by: Ln(C/C_0_) = −Kt, to calculate the mRNA half-life (t1/2). When 50% of mRNA is decayed (C/C_0_ = 1/2), the equation was: Ln (1/2) = −Kt_1/2_.

### 2.10. m^6^A Site Validation

The peak obtained by sequencing was used to predict the m^6^A methylation site by the software SRAMP (a sequence-based N6-methyladenosine (m^6^A) modification site predictor). According to the difference in the transcription of m^6^A methylation sites by the BstI enzyme, reverse transcription primers were designed, and RT-qPCR site verification was performed [28].

### 2.11. Western Blotting

The total protein of the sample was extracted using a RIPA Lysis Buffer (Strong) kit, and a gel was prepared with an SDS–PAGE Gel Kit. After SDS–PAGE, the bands were transferred to a PVDF membrane, and Western blotting was carried out according to the One Step Western Kit HRP kit, and then we used the eECL Western Blot Kit to prepare ECL chemiluminescence working solution to detect the stained membrane. The antibody used for Western blotting was an anti-EGFP antibody (mouse origin).

### 2.12. Dual-Luciferase Reporter Assay

*Cyp307a1* and *Cyp18a1* of both the wild-type and mutant sequences (A-T) containing the m^6^A methylation modification site were cloned into the *Nco*I site of the pGL-*A3*-*luc*-sv40 plasmid to construct the plasmids pGL-*A3*-*Cyp307a1*-*wt/mut*-*luc*-sv40 and pGL-*A3*-*Cyp18A1*-*wt/mut*-*luc*-sv40, respectively. BmN cells were seeded in triplicate in 24-well plates and infected with the BmBacJS13-*YTHDF3* virus. After 24 h, according to the instructions of the Lipofectamine 2000 kit (Invitrogen), pGL-*A3*-*Cyp307a1*-*wt/mut*-*luc*-sv40, pGL-*A3*-*Cyp18a1*-*wt/mut*-*luc*-sv40 and RPL-CMV were transfected at a ratio of 2:1. The cells were incubated for 72 h, and firefly luciferase (Fluc) and Renilla luciferase (Rluc) activities were measured using the Dual-Luciferase Reporter Assay System (Promega) according to the supplier’s instructions and calculated by dividing Fluc by the Rluc relative luciferase activity. The lysate remaining from the luciferase activity measurement was cleaved with TRIzol reagent, and total RNA was extracted for the qPCR analysis of the *Cyp307a1* and *Cyp18a1* mRNA abundance.

### 2.13. Statistical Analysis

SPSS 22.0 software was used for the analysis of the significant difference between treatments. Statistical comparisons were performed by using *t*-tests (two tailed) as indicated in the figure legends. The data are presented as the mean ± standard deviation (SD). The * represents *p* ≤ 0.05 significant difference and ** represents *p* ≤ 0.01 extremely significant difference. All experiments were reproduced at least three times in separate and independent replicates.

## 3. Results

### 3.1. Knockdown or Overexpression of YTHDF3 in BmN Cells

**Figure 1:**
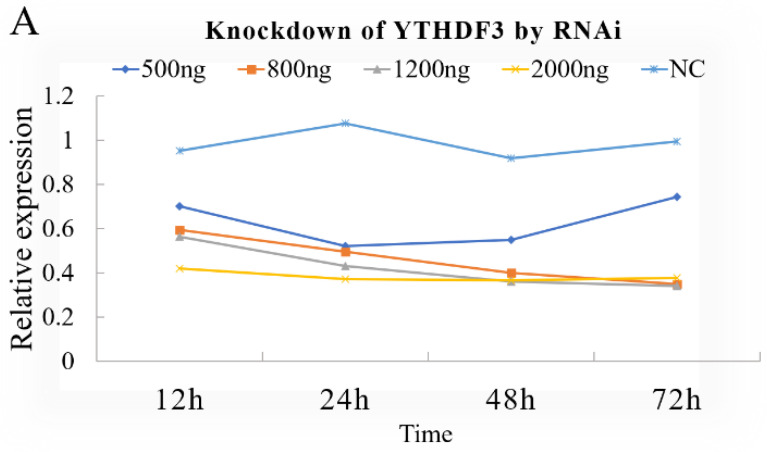

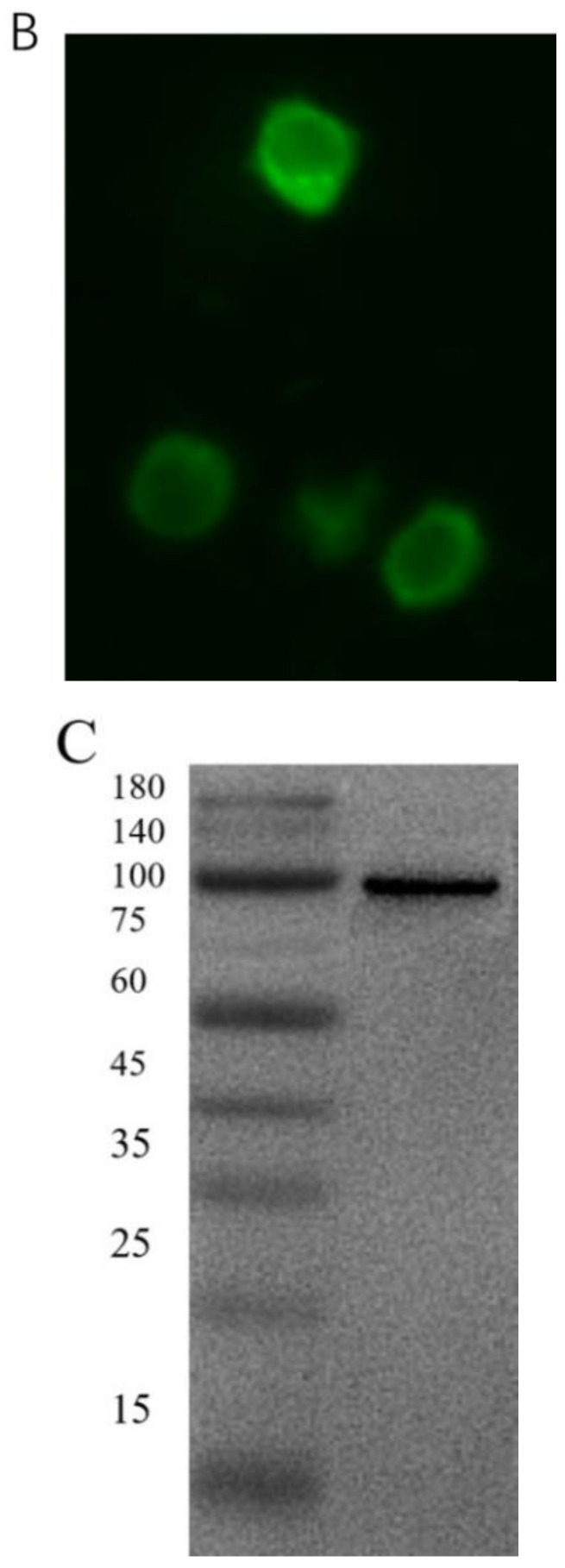
Knockdown and overexpression of YTHDF3 in BmN cells. (**A**) Knockdown of YTHDF3 by RNAi; (**B**) Overexpression and localization of BmBacJS13-*egfp*-*YTHDF3* in BmN cells; (**C**) Western blotting of BmBacJS13-*egfp*-*YTHDF3*.

To verify the effect and efficiency of the synthetic dsRNA, the synthetic dsRNA was transfected into cells at a rate of 500 ng, 800 ng, 1200 ng and 2000 ng per well. At 12 h, 24 h, 48 h and 72 h post-transfection, the cells were collected for qPCR. The results showed that the expression of YTHDF3 in cells was down-regulated at each concentration, indicating that dsYTHDF3 has an interfering effect. Additionally, the best efficiency was obtained at 48 h at 800 ng, the expression decreased by about 2.5-fold (Figure 1A).

The constructed recombinant vector was transfected into cells. Four days later, cells were observed to express fluorescent proteins, indicating that the vector construct was successfully expressed and the protein was localized in the cytoplasm of BmN cell (Figure 1B), reconfirming the conclusion that the YTHDF3 gene plays a regulatory function in the cytoplasm. Then, the cells were centrifuged at 4000 rpm for 30 min, and the supernatant and precipitate were collected for subsequent infection experiments and the identification of fusion protein expression with an EGFP antibody. Western blot analysis showed that the size of the recombinant virus BmBacJS13-*egfp*-*YTHDF3* was 101 kDa (Figure 1C), which was consistent with the predicted results, revealing that the vector was successfully constructed and could be expressed in BmN cells.

### 3.2. YTHDF3 Regulates B. mori Diapause

**Figure 2:**
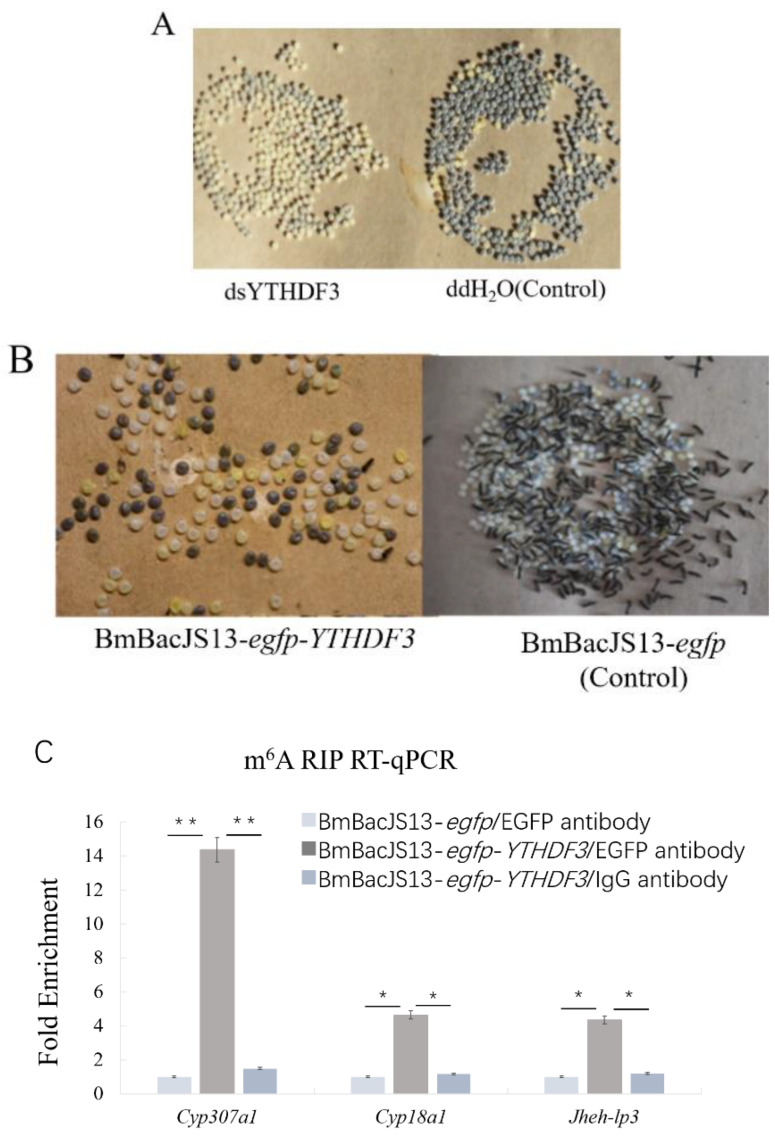
YTHDF3 regulates *B.* *mori* diapause. (**A**) Silkworm egg development is delayed after YTHDF3 knockdown; (**B**) Overexpression of YTHDF3 induces diapause; (**C**) After overexpressing YTHDF3 and egfp, total RNA was subjected to m^6^A RIP followed by RT-qPCR. Values are mean ± s.d. of n = 4 independent experiments. * *p* < 0.05; ** *p* < 0.01; two tailed Student’s *t*-test.

To explore the effect of the YTHDF3 gene on silkworm diapause, dsRNA or recombinant virus was injected into QFLT pupae on the second day and maintained at 25 °C until eclosion. The moths mated and the phenotypes of the eggs laid by the treated female moths were observed. The results showed that the pupae of the experimental group and the control group transformed into moths on the same day, but the eggs laid by moths from the YTHDF3 knockdown group hatched one day after the control group (Figure 2A). In the YTHDF3-overexpressing group, eggs were a mixture of diapause (48.2%, 66) and nondiapause (51.8%, 71), of which the nondiapause eggs hatched after incubating for 9 days at 25 °C, whereas diapause eggs remained in diapause state for about six months without hatching (Figure 2B). These results indicate that YTHDF3 has a regulatory effect on the formation of diapause in bivoltine silkworm strains.

### 3.3. Identification of Target Genes in the Ecdysone Synthesis Pathway Regulated by YTHDF3

YTHDF3 is a methylation reader and its biological function is mainly to regulate the stability and translation of m^6^A methylation-modified genes. Based on the previous research results of predecessors and our laboratory, we speculate that the effect of silkworm YTHDF3 on diapause traits may be achieved through the regulation of its target genes. According to the MeRIP-seq analysis, there are multiple genes modified by m^6^A methylation in the ecdysone synthesis pathway. Genes with m^6^A modification were predicted by software (*cat*RAPID) with YTHDF3, and the three genes *Cyp307a1*, *Jheh-lp3* and *Cyp18a1* showed potential binding sites. Then, the peak was used to predict the methylation site using the software SRAMP, and it was found that the modified sites were highly consistent with the YTHDF3 binding sequence. Therefore, after overexpressing BmBacJS13-*egfp* and BmBacJS13-*egfp*-*YTHDF3* in BmN cells, RIP was performed with EGFP antibody or a control IgG antibody, and RT-qPCR analysis was performed. The results showed that the binding amounts of *Cyp307a1*, *Cyp18a1* and *Jheh-lp3* to the BmBacJS13-*egfp*-*YTHDF3*/EGFP antibody were significantly higher than those to the control BmBacJS13-*egfp*/EGFP antibody and BmBacJS13-*egfp*-*YTHDF3*/IgG antibody (Figure 2C), indicating that YTHDF3 can recognize and bind to the m^6^A modification site of *Cyp307a1*, *Cyp18a1* and *Jheh-lp3*. Among them, *Cyp307a1* and *Cyp18a1* are involved in the synthesis of ecdysone in the hormone synthesis pathway. *Cyp307a1* can promote the expression of 20-hydroxyecdysone, and 20-hydroxyecdysone can also promote the expression of *Cyp18a1*, which ultimately promotes the expression of 20,26-dihydroxyecdysone.

### 3.4. YTHDF3 Mediates Cyp307a1 mRNA Degradation in the Ecdysone Pathway

**Figure 3:**
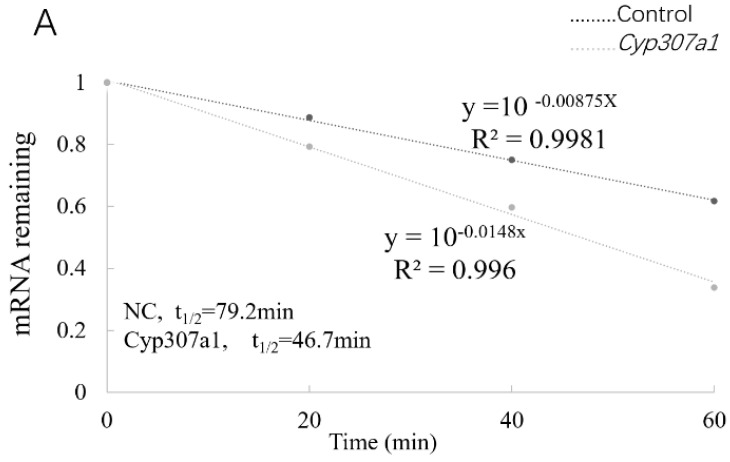

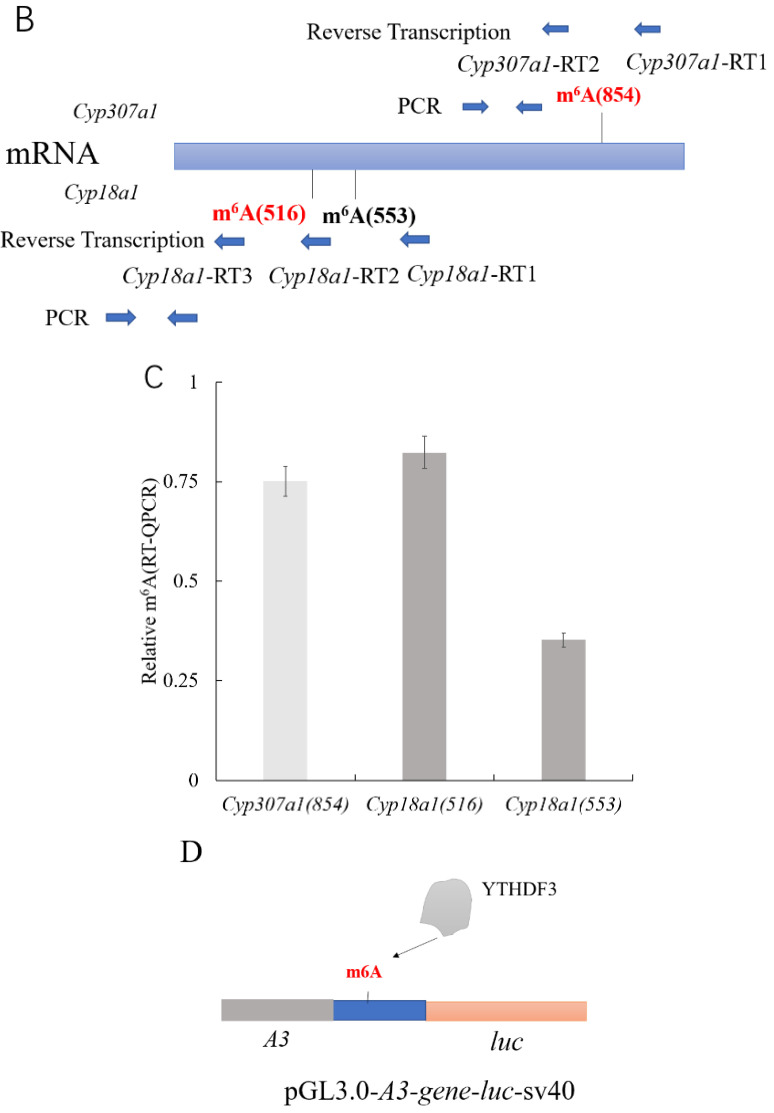

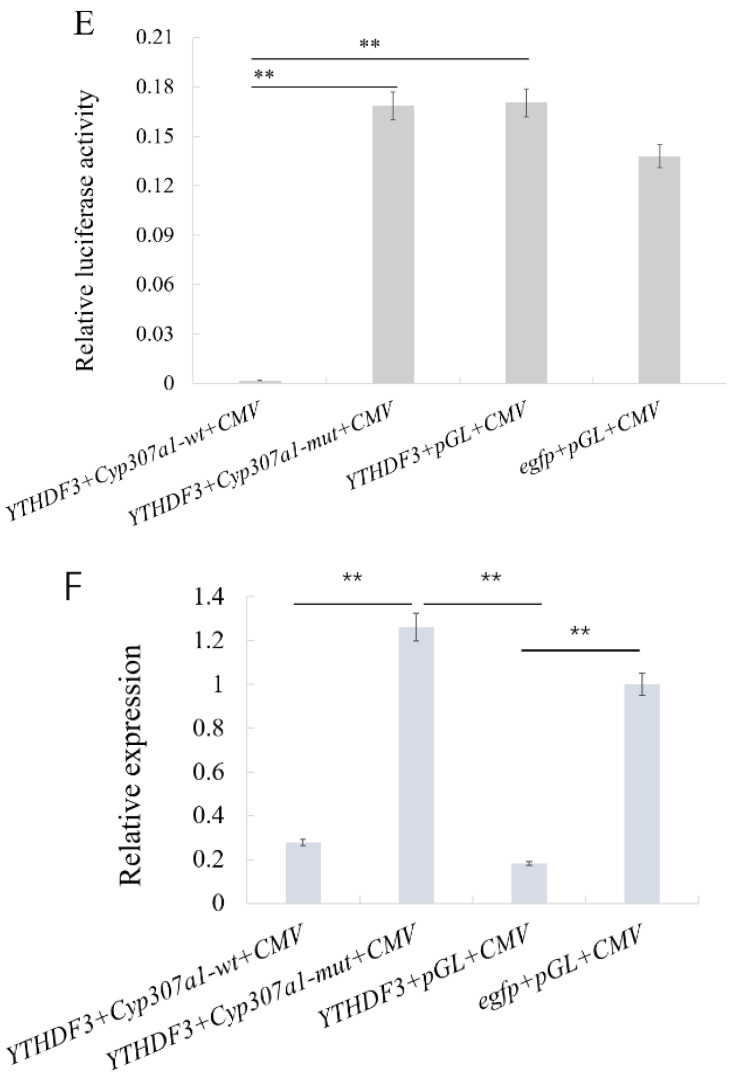
YTHDF3 mediates *Cyp307a1* mRNA degradation in the ecdysone pathway. (**A**) Reducing *Cyp307a1* mRNA half-life by overexpressing YTHDF3 in BmN cells. (**B**) Graphical representation of retrotranscription followed by PCR of the m^6^A site of *Cyp307a1* and *Cyp18a1*. (**C**) Relative quantification of the m^6^A levels of *Cyp307a1* and *Cyp18a1* in BmN cells. Relative RT-QPCR data correspond to the mean and standard error of three independent experiments. (**D**) Construction of the dual-luciferase reporter assay. (**E**) Relative firefly luciferase (Fluc) activity. (**F**) mRNA level of *Cyp307a1*. Values are mean ± s.d. of n = 4 independent experiments. Two-tailed Student’s *t*-tests were used (** *p* < 0.01).

To explore how YTHDF3 regulates the expression of *Cyp307a1*, the stability of *Cyp307a1* mRNA was tested after the overexpression of YTHDF3 in the cells and then treatment with actinomycin D. The results showed that compared with the control group, the half-life of *Cyp307a1* in the experimental group was significantly shortened, indicating that YTHDF3 could affect the stability of *Cyp307a1* mRNA and accelerate the degradation of the molecule (Figure 3A). To further investigate whether m^6^A mediates the regulation of *Cyp307a1* by YTHDF3, RT-qPCR verification was performed with the primers listed in Table 1. The results showed that there is a methylation modification at site A (854), which is the binding region of YTHDF3 (Figure 3B,C). Then, we designed wild-type (wt) and mutant (A-T) primers for this site and amplified the correct sequences to construct a dual-luciferase gene reporter plasmid (Table 1 and Figure 3D). The constructed wild-type, mutant and blank control plasmids were transfected into BmN cells. The results showed that the luciferase activity of wt *Cyp307a1* was significantly reduced compared with that of the three different controls, which indicated that YTHDF3 could regulate the expression of *Cyp307a1* and ruled out the effect of the EGFP on *Cyp307a1* (Figure 3E). The quantitative results showed that after the overexpression of YTHDF3, the mRNA expression of *Cyp307a1* in the wt group and pGL-*A3*-*luc*-sv40 group was significantly reduced, while the expression of *Cyp307a1* in the mutant group increased (Figure 3F). In conclusion, YTHDF3 can recognize and bind to the m^6^A site of *Cyp307a1*, which reduces the stability of *Cyp307a1* and inhibits the translation of the latter, but it cannot bind to *Cyp307a1* after m^6^A site mutation.

### 3.5. YTHDF3 Mediates Cyp18a1 mRNA Degradation in the Ecdysone Synthesis Pathway

**Figure 4:**
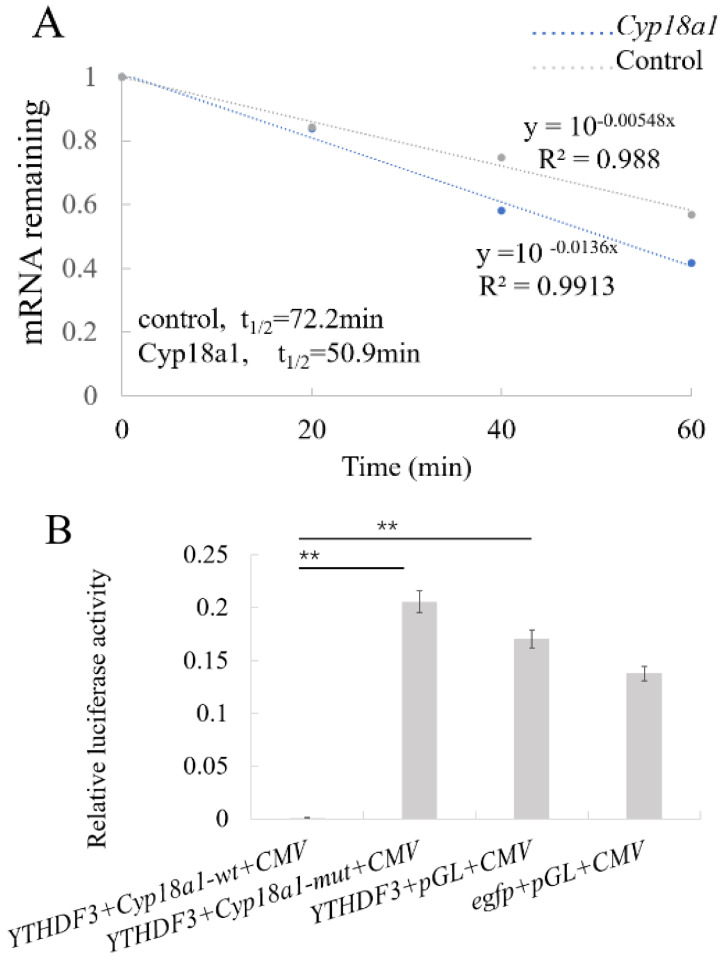

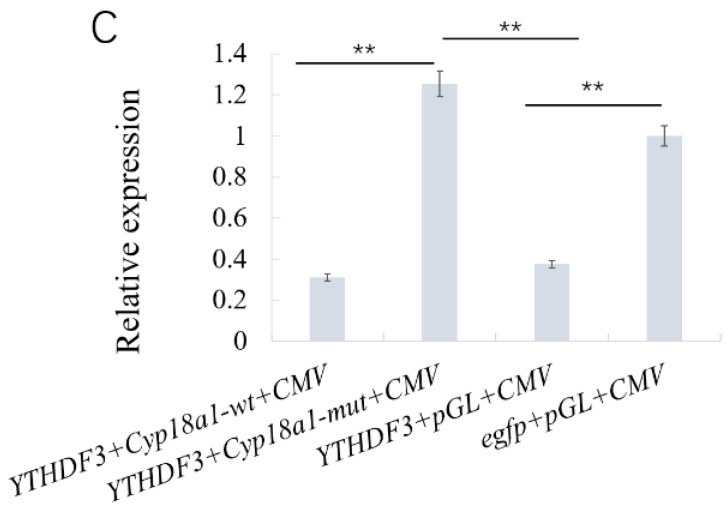
YTHDF3 mediates *Cyp18a1* mRNA degradation in the ecdysone pathway. (**A**) Reducing the *Cyp18a1* mRNA half-life by overexpressing YTHDF3 in BmN cells. (**B**) Relative firefly luciferase activity. (**C**) mRNA level of *Cyp18a1*. Values are mean ± s.d. of n = 4 independent experiments. Two-tailed Student’s t-tests were used (** *p* < 0.01).

Similarly, to explore the regulatory effect of YTHDF3 on the P450 gene, the stability of *Cyp18a1* was tested, and the results showed that the half-life of *Cyp18a1* mRNA is significantly reduced, which indicates that YTHDF3 also reduces the stability of *Cyp18a1* (Figure 4A). The software predicted that there are two methylation modification sites in the fragment and designed the methylation site primers (Table 1). The RT-qPCR verification results showed that A (513) was a methylation modification site (Figure 3B,C). After the modification sites were identified, double luciferase reporter plasmids for m^6^A wild type and A-T mutant were constructed according to the schematic diagram in Figure 3D. The plasmids were also transfected into BmN cells for luciferase activity detection. The results showed that the luciferase activity of wild-type *Cyp18a1* was significantly lower than that of the mutant *Cyp18a1* and the two control groups (Figure 4B). The RT-qPCR results showed that compared with the BmBacJS13-*egfp* + pGL-*A3*-*luc*-sv40 group, the mRNA expression of the *Cyp18a1* gene in the wild-type group and the BmBacJS13-*egfp* + pGL-*A3*-*luc*-sv40 group was significantly decreased, while mutant *Cyp18a1* expression was increased (Figure 4C). The results indicated that YTHDF3 could recognize *Cyp18a1* and reduce mRNA stability.

## 4. Discussion

Epigenetic modification is an important biological regulatory mechanism for diapause traits in insects and is involved in the regulation of various diapause types, such as pupal diapause and adult diapause [29,30], but the molecular mechanism behind its function is unclear. Studies have reported that the expression changes in YTHDC1 and YTHDF3 in the pupal ovaries of different *B. mori* are quite different, indicating that m^6^A modification in silkworm may regulate the expression of diapause-related genes [27]. Therefore, we propose that the bivoltine silkworm YTHDF3 affects the diapause of silkworm eggs by regulating the expression of diapause-related genes. In this study, we carried out YTHDF3 knockdown and overexpression experiments in the bivoltine silkworm and found that YTHDF3 knockdown in *B. mori* resulted in delayed embryo (egg) development. Overexpression of YTHDF3 resulted in the transformation of nondiapause-destined eggs into a mixture of diapause and nondiapause eggs, confirming the involvement of YTHDF3 in the regulation of bivoltine silkworm diapause. While the results showed that YTHDC1 was localized in the nucleus, as in other species [31], our knockdown and overexpression experiments showed no significant effect on the diapause phenotype (data not shown). As a reader protein, YTHDF3 functions to exert regulatory functions by affecting gene expression [32]. Combined with MeRIP-seq sequencing, we screened three methylated genes related to diapause and further verified that the methylation sites of *Cyp307a1* and *Cyp18a1* in ESP can be recognized and bound by YTHDF3. An increase in *Cyp307a1* can promote the expression of 20E, and *Cyp18a1* is a downstream gene of 20E and affects the expression of the 20,26-dihydroxyecdysone gene, both of which are important genes in the ESP and are important hormones affecting diapause traits [33,34,35,36]. Our experimental results show that YTHDF3 reduces the mRNA stability of *Cyp307a1* and *Cyp18a1* and inhibits translation, thereby affecting the diapause traits of silkworm eggs.

*Cyp307a1* and *Cyp18a1* are two target genes of YTHDF3, but not all target genes of YTHDF3 affect diapause. Our experimental results show that *Jheh-lp3*, an important gene in the juvenile hormone pathway [37], can also be recognized by YTHDF3, but further experiments showed that YTHDF3 has no obvious regulatory effect on it (data not shown). Under the influence of temperature, light and other environmental factors, some regulatory network changes occurred in the bivoltine silkworm strain eggs, which were associated with a large number of diapause-related genes [38]. In this study, only two genes in the ESP were involved. YTHDF3 probably also regulates other target genes that may have regulatory effects on diapause.

Functional studies have shown that YTHDF family genes mainly regulate mRNA stability and translation [6,32]. It has also been reported that YTHDF1/2/3 paralogues are highly similar in sequence, functional domains, interacting proteins and intracellular localization [39,40]. The different knockout patterns of YTHDF1/2/3 have shown different effects on mouse embryonic death efficiency, and there is a dose-dependent redundancy among YTHDF members [15]. Therefore, the role of each YTHDF is currently not fully understood. As the sole member of the YTH family, YTHDF3 in silkworm may undertake more regulatory functions. The expression of YTHDF3 mediated by siRNA can inhibit the infection efficiency of *B. mori* nuclear polyhedrosis virus (BmNPV) [41]. Decreased METTL3 expression can inhibit the proliferation of BmNPV, suggesting that the reader regulates the expression of viral proliferation-related genes mediated by m^6^A modification [42]. To date, research reports on the involvement of RNA modification in the regulation of insect diapause are very limited, which may be related to the lack of powerful methylation systems in diapause model insects; for example, no demethylase has been found in silkworm. However, m^6^A is one of the most abundant chemical modifications on mRNAs, its importance to gene expression regulation is self-evident, and it is also a new entry point for the study of diapause network regulation.

At present, most of the research is on the enzymes involved in the methylation modification process and the changes in the transcript abundance of the modified genes, but the functional relevance of these changes is largely unknown. Genetic modification is a systematic regulation process that has different biological functions in each developmental stage. Whether these modifications are related to the regulation of genes at the three levels of DNA, RNA and protein must be further studied. Moreover, the regulatory mechanism of how these modifications regulate gene expression levels to affect diapause is unclear and requires further study. Therefore, taking epigenetics as an entry point, it is worth exploring how epigenetics responds to environmental signals during the embryonic period of the bivoltine silkworm and the molecular regulatory mechanisms involved in their diapause traits.

## 5. Conclusions

Our study shows that m^6^A modification mediates YTHDF3 to affect the expression levels of its target genes, *Cyp307a1* and *Cyp18a1*, in the ESP to regulate diapause in bivoltine *B. mori*. This thesis has provided a deeper insight into the epigenetic regulation mechanism of diapause traits in the bivoltine silkworm, and also provides new experimental data for elucidating the diapause regulatory network. Future experiments will be devoted to the epigenetic mechanisms of molecular regulation in response to environmental cues in embryonic diapause, which will be a fruitful area for further work.

## Figures and Tables

**Table 1:** Primers.

Primer Name	Sequence	Primer Purpose
*egfp-F*	ATGGTGAGCAAGGGC	pFastBac Dual
*egfp-R*	CTTGTACAGCTCGTCC	pFastBac Dual
*YTHDF3-F*	ATGTCAGCAGGCGTGTCAG	pFastBac Dual
*YTHDF3-R*	TTAATTGCGGGGACGTCCTCG	pFastBac Dual
*YTHDF3T7-F*	GGATCCTAATACGACTCACTATAGGTCGCAACTATCGTGAGCATC	dsRNA
*YTHDF3-R*	TGCATTTTCTGATTCCCCTC	dsRNA
*YTHDF3-F*	TCGCAACTATCGTGAGCATC	dsRNA
*YTHDF3T7-R*	GGATCCTAATACGACTCACTATAGGTGCATTTTCTGATTCCCCTC	dsRNA
*YTHDF3-F*	ATCAGCGGATGAAAGGGCAA	Real-time PCR
*YTHDF3-R*	AGGCGCATAAGAAGGTTGCT	Real-time PCR
*Cyp307a1-RT1*	CGTGTAGCACCCTGAGGAGGCC	Reverse Transcription
*Cyp307a1-RT2*	CCGGAGCTTCCGTGTCTAAGC	Reverse Transcription
*Cyp307a1-F*	TGGGAGATCAACCAGGGCTA	Real-time PCR
*Cyp307a1-R*	GAAGGATCGGATGTCCTGGG	Real-time PCR
*Cyp18a1-RT1*	CCTCGTCCTTCCATGATTTCG	Reverse Transcription
*Cyp18a1-RT2*	GGTCTCGCGATAGAATCCGAT	Reverse Transcription
*Cyp18a1-RT3*	TCGGCGAATTGAGACAGACGA	Reverse Transcription
*Cyp18a1-F*	TGTGGACGAAACTCGACACG	Real-time PCR
*Cyp18a1-R*	AAACGGTACACCCCATGGTC	Real-time PCR
*Cyp307a1-wtF*	AGAAGGACTTTCTGGACGGC	pGL-A3-luc
*Cyp307a1-mutF*	AGAAGGTCTTTCTGGACGGC	pGL-A3-luc
*Cyp307a1-R*	TACGGGTCTTTTGCCTCTGG	pGL-A3-luc
*Cyp18a1-wtF*	CGCCGAAACTTGTGAAGGAA	pGL-A3-luc
*Cyp18a1-mutF*	CGCCGAAACTTGTGAAGGAT	pGL-A3-luc
*Cyp18a1-R*	CGGCCAAACCTTGACTCCAA	pGL-A3-luc

## Data Availability

All data are contained within the article.

## References

[1] Li H.-B., Tong J., Zhu S., Batista P.J., Duffy E.E., Zhao J., Bailis W., Cao G., Kroehling L., Chen Y. (2017). m6A mRNA methylation controls T cell homeostasis by targeting the IL-7/STAT5/SOCS pathways. Nature.

[2] Taketo K., Konno M., Asai A., Koseki J., Toratani M., Satoh T., Doki Y., Mori M., Ishii H., Ogawa K. (2018). The epitranscriptome m6A writer METTL3 promotes chemo- and radioresistance in pancreatic cancer cells. Int. J. Oncol..

[3] Hou G., Zhao X., Li L., Yang Q., Liu X., Caihu H., Runhui L., Chen R., Wang Y., Jiang B. (2021). SUMOylation of YTHDF2 promotes mRNA degradation and cancer progression by increasing its binding affinity with m6A-modified mRNAs. Nucleic Acids Res..

[4] Dominissini D., Moshitch-Moshkovit S., Schwartz S., Salmon-Divon M., Ungar L., Osenberg S., Cesarkas K., Jacob-Hirsch J., Amariglio N., Kupiec M. (2012). Topology of the human and mousem^6^A RNA methylomes revealed by m^6^A-seq. Nature.

[5] Slobodin B., Han R., Calderone V., Vrielink J.A.F.O., Loayza-Puch F., Elkon R., Agami R. (2017). Transcription Impacts the Efficiency of mRNA Translation via Co-transcriptional N6-adenosine Methylation. Cell.

[6] Wang X., Zhao B.S., Roundtree I.A., Lu Z., Han D., Ma H., Weng X., Chen K., Shi H., He C. (2015). N6-methyladenosine Modulates Messenger RNA Translation Efficiency. Cell.

[7] Du H., Zhao Y., He J., Zhang Y., Xi H., Liu M., Ma J., Wu L. (2016). YTHDF2 destabilizes m6A-containing RNA through direct recruitment of the CCR4–NOT deadenylase complex. Nat. Commun..

[8] Alarcón C.R., Goodarzi H., Lee H., Liu X., Tavazoie S., Tavazoie S.F. (2015). HNRNPA2B1 Is a Mediator of m6A-Dependent Nuclear RNA Processing Events. Cell.

[9] Liu N., Dai Q., Zheng G., He C., Parisien M., Pan T. (2015). N(6)-methyladenosine-dependent RNA structural switches regulate RNA-protein interactions. Nature.

[10] Huang H., Weng H., Sun W., Qin X., Shi H., Wu H., Zhao B.S., Mesquita A., Liu C., Yuan C.L. (2018). Recognition of RNA N(6)-methyladenosine by IGF2BP proteins enhances mRNA stability and translation. Nat. Cell Biol..

[11] Lacerda R., Menezes J., Romão L. (2017). More than just scanning: The importance of cap-independent mRNA translation initiation for cellular stress response and cancer. Cell Mol. Life Sci..

[12] Xu C., Wang X., Liu K., Roundtree I.A., Tempel W., Li Y., Lu Z., He C., Min J. (2014). Structural basis for selective binding of m6A RNA by the YTHDC1 YTH domain. Nat. Chem. Biol..

[13] Wang X., Lu Z., Gomez A., Hon G.C., Yue Y., Han D., Fu Y., Parisien M., Dai Q., Jia G. (2014). N6-methyladenosine-dependent regulation of messenger RNA stability. Nature.

[14] Shi H., Wang X., Lu Z., Zhao B.S., Ma H., Hsu P.J., Liu C., He C. (2017). YTHDF3 facilitates translation and decay of N6-methyladenosine-modified RNA. Cell Res..

[15] Lasman L., Krupalnik V., Viukov S., Mor N., Aguilera Castrejon A., Schneir D., Bayerl J., Mizrahi O., Peles S., Tawil S. (2020). Context-dependent functional compensation between Ythdf m6A reader proteins. Genes Dev..

[16] Lennon J.T., Jones S.E. (2011). Microbial seed banks: The ecological and evolutionary implications of dormancy. Nat. Rev. Microbiol..

[17] Boon C., Li R., Qi R., Dick T. (2001). Proteins of Mycobacterium bovis BCG induced in the Wayne dormancy model. J. Bacteriol..

[18] Saunders D. (1977). Insect Clocks. Comparative Biochemistry and Physiology Part A: Physiology.

[19] Bonasio R., Tu S., Reinberg D. (2010). Molecular signals of epigenetic states. Science.

[20] Pegoraro M., Bafna A., Davies N.J., Shuker D.M., Tauber E. (2016). DNA methylation changes induced by long and short photoperiods in Nasonia. Genome Res..

[21] Delaney C.E., Chen A.T., Graniel J.V., Dumas K.J., Hu P.J. (2017). A histone H4 lysine 20 methyltransferase couples environmental cues to sensory neuron control of developmental plasticity. Development.

[22] George S., Palli S.R. (2020). Histone deacetylase 3 is required for development and metamorphosis in the red flour beetle, Tribolium castaneum. BMC Genom..

[23] Lyu H., Xu G., Chen P., Song Q., Feng Q., Yi Y., Zheng S. (2020). 20-Hydroxyecdysone receptor-activated Bombyx mori CCAAT/enhancer-binding protein gamma regulates the expression of BmCBP and subsequent histone H3 lysine 27 acetylation in Bo. mori. Insect Mol. Biol..

[24] Hůla P., Kostál V. Potential role for histone H3K4 methylation in diapause induction in the larva of Chymomyza costata. Proceedings of the 7th International Symposium of the Environmental Physiology of Ectotherms and Plants.

[25] Lekha G., Gupta T., Awasthi A.K., Murthy G.N., Trivedy K., Ponnuvel K.M. (2015). Genome wide microarray based expression profiles associated with BmNPV resistance and susceptibility in Indian silkworm races of Bombyx mori. Genomics.

[26] Li B., Hu P., Zhang S.Z., Toufeeq S., Wang J., Zhao K., Xu X., Xu J.P., Huang S.J. (2019). DNA methyltransferase *BmDnmt1* and *BmDnmt2* in silkworm (*Bombyx mori*) and the regulation of silkworm embryonic development. Arch. Insect Biochem. Physiol..

[27] Jiang T., Li J., Qian P., Xue P., Xu J., Chen Y., Zhu J., Shunming T., Zhao Q., Qian H. (2019). The role of N6-methyladenosine modification on diapause in silkworm (*Bombyx mori*) strains that exhibit different voltinism. Mol. Reprod. Dev..

[28] Castellanos-Rubio A., Santin I., Olazagoitia-Garmendia A., Romero-Garmendia I., Jauregi-Miguel A., Legarda M., Bilbao J.R. (2019). A novel RT-QPCR-based assay for the relative quantification of residue specific m6A RNA methylation. Sci. Rep..

[29] Reynolds J.A., Verlinden H. (2017). Chapter Five—Epigenetic Influences on Diapause. Advances in Insect Physiology.

[30] Santos P.K.F., de Souza Araujo N., Françoso E., Zuntini A.R., Arias M.C. (2018). Diapause in a tropical oil-collecting bee: Molecular basis unveiled by RNA-Seq. BMC Genom..

[31] Xiao W., Adhikari S., Dahal U., Chen Y., Hao Y.J., Sun B.F., Sun H.Y., Li A., Ping X., Lai W. (2016). Nuclear m6A Reader YTHDC1 Regulates mRNA Splicing. Mol. Cell.

[32] Li A., Chen Y.-S., Ping X.-L., Yang X., Xiao W., Yang Y., Sun H.-Y., Zhu Q., Baidya P., Wang X. (2017). Cytoplasmic m6A reader YTHDF3 promotes mRNA translation. Cell Res..

[33] Guittard E., Blais C., Maria A., Parvy J.-P., Pasricha S., Lumb C., Lafont R., Daborn P.J., Dauphin-Villemant C. (2011). CYP18A1, a key enzyme of Drosophila steroid hormone inactivation, is essential for metamorphosis. Dev. Biol..

[34] Namiki T., Niwa R., Sakudoh T., Shirai K., Takeuchi H., Kataoka H. (2005). Cytochrome P450 CYP307A1/Spook: A regulator for ecdysone synthesis in insects. Biochem. Biophys. Res. Commun..

[35] Kadono-Okuda K., Amornsak W., Yamashita O. (1994). Controlled Ecdysteroid Accumulation in Eggs of the Silkworm, *Bombyx mori*, by an lmidazole Compound (KK-42), and Embryogenesis in These Eggs. Arch. Insect Biochem. Physiol..

[36] Haruyuki S., Hideki T., Toshihiro M., Hibiki T., Noriyuki H., Yoshinori F. (1999). Comparative Studies of Ecdysteroid Metabolism between Diapause Eggs and Non-diapause Eggs of the Silkworm, *Bombyx mori*. Zool. Sci..

[37] Seino A., Ogura T., Tsubota T., Shimomura M., Nakakura T., Tan A., Mita K., Shinoda T., Nakagawa Y., Shiotsuki T. (2010). Characterization of Juvenile Hormone Epoxide Hydrolase and Related Genes in the Larval Development of the Silkworm *Bombyx mori*. Biosci. Biotechnol. Biochem..

[38] Chen Y.R., Jiang T., Zhu J., Xie Y.C., Tan Z.C., Chen Y.H., Tang S.M., Hao B.F., Wang S.P., Huang J.S. (2017). Transcriptome sequencing reveals potential mechanisms of diapause preparation in bivoltine silkworm *Bombyx mori* (Lepidoptera: Bombycidae). Comp. Biochem. Physiol. Part D Genom. Proteom..

[39] Zaccara S., Jaffrey S.R. (2020). A Unified Model for the Function of YTHDF Proteins in Regulating m6A-Modified mRNA. Cell.

[40] Patil D.P., Pickering B.F., Jaffrey S.R. (2018). Reading m6A in the Transcriptome: m6A-Binding Proteins. Trends Cell Biol..

[41] Zhang X., Zhang Y., Dai K., Liang Z., Zhu M., Pan J., Zhang M., Yan B., Zhu H., Zhang Z. (2019). N (6)-Methyladenosine Level in Silkworm Midgut/Ovary Cell Line Is Associated with Bombyx mori Nucleopolyhedrovirus Infection. Front. Microbiol..

[42] Xue P., Jiang T., Zhu J., Wang M., Zhao Q., Huang J., Tang S., Shen X. (2021). Low METTL3 level in midgut of the Bombyx mori inhibit the proliferation of nucleopolyhedrovirus. J. Asia-Pac. Entomol..

